# 10080 Quantifying type of dental treatment rendered for patients with special needs

**DOI:** 10.1017/cts.2021.695

**Published:** 2021-03-30

**Authors:** Sydnee Chavis

**Affiliations:** University of Maryland School of Dentistry

## Abstract

ABSTRACT IMPACT: Quantifying the types of dental procedures patients with healthcare needs receive can help understand and improve modalities of dental care to improve healthcare outcomes. OBJECTIVES/GOALS: 1. Quantify how medical complexity based on special needs diagnosis impacts dental treatment rendered

2. Comprehend how medical diagnosis of a special need can affect rate of treatment and type of treatment METHODS/STUDY POPULATION: This study consists of a chart review of all active patients in a dental school setting who have one of the following diagnoses of a special need: autism, developmental disorder, epilepsy, cerebral palsy, neuromuscular disorder, and hydrocephaly. Medical diagnoses were used to extract records and quantify the types of dental treatment rendered for these patients (preventative, restorative, and surgical), as well as the rates of appointments for this patient population. RESULTS/ANTICIPATED RESULTS: The medical complexity of patients of this population, as defined by the number medical diagnoses, impacts the type of treatment rendered as well as how frequently these patients are seen for dental care (rate of appointments). DISCUSSION/SIGNIFICANCE OF FINDINGS: Over- or under-utilization of dental treatment modalities can impact the oral health status and outcomes for patients with special needs. The differences in types and frequency/ rate of dental treatment rendered for patients of different medical complexity can further impede their oral health and systemic health status and health outcomes.

